# Dysphagia Prevalence and Outcomes Associated with the Evolution of COVID-19 and Its Variants in Critically Ill Patients

**DOI:** 10.1007/s00455-023-10598-7

**Published:** 2023-06-22

**Authors:** Nicola A. Clayton, Amy Freeman-Sanderson, Elizabeth Walker

**Affiliations:** 1 Speech Pathology Department & Intensive Care Unit, Concord Repatriation General Hospital, Building 42, Hospital Rd, Concord, Sydney, NSW 2139 Australia; 2 Faculty of Medicine and Health, The University of Sydney, Sydney, NSW Australia; 3 School of Health & Rehabilitation Sciences, The University of Queensland, Brisbane, QLD Australia; 4 Speech Pathology Department & Intensive Care Unit, Royal Prince Alfred Hospital, Sydney, NSW Australia; 5 Graduate School of Health, University of Technology Sydney, Sydney, NSW Australia; 6 Critical Care Division, The George Institute for Global Health, Sydney, NSW Australia; 7 Australian and New Zealand Intensive Care Research Centre (ANZIC-RC), School of Public Health and Preventive Medicine, Monash University, Melbourne, VIC Australia

**Keywords:** COVID-19, SARS-CoV2, Dysphagia, Critically ill, Recovery

## Abstract

Data collected during the 2020–21 COVID-19 alpha wave indicated dysphagia prevalence rates up to 93%. Whilst many patients recovered during hospital admission, some experienced persistent dysphagia with protracted recovery. To explore (1) prevalence, (2) treatment, and (3) recovery patterns and outcomes for swallowing, in the ICU patient with Delta and subsequent variants of COVID-19. Prospective observational study. Patients admitted to 26 Intensive Care Units (ICUs) over 12 months, diagnosed with COVID-19, treated for survival and seen by Speech–Language Pathology (SLP) for clinical swallowing assessment were included. Demographic, medical, SLP treatment, and swallowing outcome data were collected. 235 participants (63% male, median age = 58 years) were recruited. Median mechanical ventilation was 16 days, and ICU and hospital length of stay (LOS) were 20 and 42 days, respectively. ICU-Acquired Weakness (54%) and delirium (49%) were frequently observed. Prevalence of dysphagia was 94% with the majority (45%) exhibiting profound dysphagia (Functional Oral Intake Scale = 1) at initial assessment. Median duration to initiate oral feeding was 19 days (IQR = 11-44 days) from ICU admission, and 24% received dysphagia rehabilitation. Dysphagia recovery by hospital discharge was observed in 71% (median duration = 30 days [IQR = 17-56 days]). Positive linear associations were identified between duration of intubation, mechanical ventilation, hospital and ICU LOS, and duration to SLP assessment (*p* = 0.000), dysphagia severity (*p* = 0.000), commencing oral intake (*p* = 0.000), dysphagia recovery (*p* < 0.01), and enteral feeding (*p* = 0.000). Whilst older participants had more severe dysphagia (*p* = 0.028), younger participants took longer to commence oral feeding (*p* = 0.047). Dysphagia remains highly prevalent in ICU COVID-19 patients. Whilst invasive ventilation duration is associated with swallowing outcomes, more evidence on dysphagia pathophysiology is required to guide rehabilitation.

## Introduction

Evidence describing the impact of COVID-19 and its variants on swallowing function continues to emerge. Literature to date indicates that following SARS-CoV-2 infection with admission to critical care, there is a high prevalence of dysphagia at initial swallow assessment, with rates ranging between 55 and 93% across various international cohorts in the Alpha wave of the pandemic [1, 2]. In line with other critically ill populations, there appears to be a clear correlation between COVID dysphagia and critical care outcomes including duration of intubation, mechanical ventilation, tracheostomy, age [1–5], and neurological manifestations experienced by affected patients [6].

Data describing the degree of dysphagia and the trajectory of recovery are vital, as this enables evidence-driven health service needs as the world continues to navigate increased healthcare burden. From critically ill cohort studies, the degree of dysphagia and its impact to function are often most severe at the point of initial speech–language pathology (SLP) consultation. Across a multi-site study in the Republic of Ireland involving 14 acute hospitals, 90% of the intubated cohort were dysphagic at initial clinical swallow assessment, with 58.6% patients requiring enteral feeding and 35.4% unable to resume any oral intake [6]. In an Australian study [2], dysphagia was again prevalent (93%) at the time of initial SLP consultation, with the majority exhibiting profound dysphagia (44%), a high dependence on enteral nutrition (100%) although a reasonable rate of complete dysphagia recovery by the time of hospital discharge (81%). Similarly, in the U.K., 87% of the ICU patients referred to SLP presented with dysphagia, with 51% unable to commence any oral intake [7]. Whilst recovery of swallowing function is expected and does occur over hospital admission, the level of recovery varies for individuals. At the point of ICU discharge, Mallart et al. [4] reported recovery rates in up to 22% of adults with dysphagia, with complete recovery as defined by resumption of an unrestricted diet. At the time of hospital discharge, persistent dysphagia rates vary from 19 to 56% [2, 6], as defined by the need for modified diet and fluids or ongoing reliance on enteral nutrition.

Management and recovery of swallowing function are underpinned by the nature and aetiology of dysphagia and its subsequent rehabilitation. Early studies examining the pathophysiology of oropharyngeal dysphagia related to SARS-CoV-2 infection postulated that causative factors are related to insults to the swallowing network across multiple domains. These specifically include both motor and sensory dysfunction across neurological, respiratory, olfactory, gustatory and laryngopharyngeal systems [8, 9]. Use of instrumental assessment to aid dysphagia diagnosis for critically ill patients with COVID has been limited, however, authors acknowledge its use can inform swallow physiology to enable diet progression and regression in the context of swallow safety [10]. Whilst access to early intervention has been recommended [11], the types and impact of compensatory and rehabilitative exercises for this clinical population are currently unknown with evidence to date focusing largely on prevalence data.

Further to this, international evidence to date has also predominantly focused on the Alpha wave of the pandemic. As restrictions and border closures have eased, the Delta, Omicron, and other variants of the SARS-CoV2 virus have breached international borders resulting in considerable increases of COVID-19 cases and subsequent hospital admissions [12]. As such, this allowed an opportunity to further investigate the impact of COVID-19 on swallowing function, the treatment that is required and subsequent patient outcomes in a larger cohort across multiple facilities.

The aims of the current study were therefore to explore (1) the prevalence, (2) treatment, and (3) recovery pattern and outcomes for swallowing, in the ICU patient with Delta and subsequent variants of COVID-19.

## Methods

This study was conducted and has been reported in accordance with the STROBE statement [13].

### Design

A multi-site prospective observational cohort study.

### Participants & Setting

All adult patients (aged 18–100 years) diagnosed with COVID-19, requiring Intensive Care Unit (ICU) admission and treated with the intent for survival across 26 participating NSW Public Hospitals (metropolitan and rural), and referred to Speech–Language Pathology (SLP) for evaluation of swallowing function during the acute hospital admission in line with site-specific referral practices, were considered for inclusion within the study. The study was conducted over a 12-month period (1st March 2021–1st March 2022).

### Demographic and Medical Outcomes

Demographic data were extracted from the medical records of all participants including age, sex, hospital length of stay (LOS) (recorded in days), and past medical history, including any pre-existing dysphagia. Data specific to the ICU were also recorded comprising ICU LOS (days), APACHE-II [14] score (medical score calculated based on how unwell a patient is at the point of ICU admission), duration of endotracheal intubation (days), duration of tracheostomy (days), duration of mechanical ventilation (days), number of intubations, medical complications, and discharge destination. All demographic and medical endpoints relating to duration were calculated from the date of admission to the ICU.

### Swallowing Outcomes

Swallowing function was assessed via SLP Clinical Swallowing Examination (CSE) with presence and severity of swallowing impairment (dysphagia) defined by the Functional Oral Intake Scale (FOIS) [15]. The FOIS [15] is 7-point numerical scale where 1 = nothing by mouth; 2 = tube dependent with minimal attempts of food and fluid; 3 = tube dependent with consistent intake of food and fluid; 4 = total oral diet of a single consistency; 5 = total oral diet with multiple consistencies but requiring special preparation or compensations; 6 = total oral with multiple consistencies without special preparation, but with specific food limitations; and 7 = total oral diet with no restriction. For the purposes of this study, dysphagia was defined as a FOIS score of 1–6. All swallowing examinations were conducted in accordance with routine clinical practice with access to modified food and commercial pre-thickened fluids to IDDSI standards [16], the clinical guidelines of Speech Pathology Australia [17] and in line with individual needs of the patients and site-specific infection control guidelines. All CSE procedures involved a comprehensive medical and swallowing case history and oromotor examination, including cranial nerve assessment, trial of food and fluids, as well as compensatory swallow strategies as clinically indicated.

Dysphagia management was considered complete once the patient had attained premorbid swallowing function ability (as determined by FOIS score) or their swallow function had stabilised such that the treating Speech–Language Pathologist had deemed that further gains were unlikely. Resolution of dysphagia was defined by the ability to consume a full oral diet and fluids without texture modification or the aid of compensatory strategies (FOIS = 7).

Further to this, a number of other specific swallowing outcomes were recorded capturing information relevant to the duration to commencing oral intake, dysphagia rehabilitation, dysphagia resolution, instrumental assessment outcomes (if conducted in line with routine clinical practice), and non-oral (enteral) feeding. All swallowing data relating to duration were calculated in days from the time of ICU admission. For those patients who underwent instrumental swallowing examination, either Videofluoroscopic Swallowing Study (VFSS) or Flexible Endoscopic Evaluation of Swallowing (FEES), in accordance with routine clinical care, additional outcome measures were employed to describe swallowing impairment.

Outcome measures applied for VFSS were the Penetration–Aspiration Scale (PAS) [18] and the Bolus Residue Scale (BRS) [19]. The PAS [18] is an 8-point scale that describes the degree of food/fluid airway invasion and airway response, where 1 = no laryngeal penetration/aspiration and 8 = aspiration below the level of the vocal folds with nil airway response. The BRS [19] is a 6-point scale which describes the degree of post-swallow pharyngeal residue, where 1 = no residue and 6 = residue in the valleculae and posterior pharyngeal wall and piriform sinus.

Outcome measures applied for FEES were the New Zealand Secretion Rating Scale (NZSS) ([20], the Penetration–Aspiration Scale [18], and the Yale pharyngeal residue severity rating scale [21]. The NZSS [20] is a 7-point scale is a 7-point scale that describes the presence and severity of secretions retained within the pharynx and larynx, where 0 = no secretions and 7 = profuse secretions being aspirated and patient unable to clear. The Yale residue scale [21] is a 5-point scale which describes the degree of post-swallow pharyngeal residue, where 1 = no residue and 5 = severe residue each at the location of the valleculae and piriform sinus.

### Data Collection

Data were collected at individual sites and subsequently inputted into a purpose-built password-protected REDCap database [22] by local site investigators via a secure survey link. A data dictionary defining each data point in addition to targeted data entry training was provided to all sites to minimise bias. Data were de-identified at the point of data entry.

The REDCap database [22] was designed so that each data field, with the exception of APACHE-II score [14], was mandatory to assist in ensuring data completeness.

### Data Analysis

Following completion of data collection, data were exported via a secure encrypted link generated by REDCap [22]. Data were subsequently downloaded into Excel and the Statistical Package for Social Sciences (SPSS Version 27.0) for analysis.

Descriptive statistics were utilised for the first stage of data analysis (*n* = 235). A conservative approach of non-normal data distribution was assumed with data reported as medians and IQR [median (IQR)]. Categorical data are presented as a proportion of the sample [n(%)]. Correlation statistics between variables were determined a priori and conducted using non-parametric assessments (Mann–Whitney *U*) between continuous and dichotomous variables, Pearson correlation between two continuous variables, and Fishers Exact Test between dichotomous variables, with statistical significance set at *p* < 0.05.

In the second stage of analysis, data from the current study’s cohort (*n* = 235) were directly compared to previously published outcomes data of a similar cohort (*n* = 27) from Clayton et al. [2]. Comparisons (Chi-square and Mann–Whitney *U*) were conducted between the current and published cohorts. Significance was set at *p* < 0.05.

This study received ethical approval (2020/ETH01301) from the CRGH Human Research & Ethics Committee. Written consent for the purposes of gathering outcomes was sought and obtained from all cases prior to data collection.

## Results

### Demographic & Critical Care Outcomes

235 patients (149 male; 86 female) with a median age of 58 years (range = 21–97 years, IQR 48–70 years) were recruited across 26 NSW public hospitals, to participate in the study. A large proportion of participants (*n* = 196; 83%) required intubation and mechanical ventilation as part of their ICU treatment, with a median intubation duration of 14 days (IQR 9–22 days). Tracheostomy placement was required in 33% (*n* = 78) with a median cannulation duration of 31 days (IQR 21–49 days). APACHE-II score was collected for 91 participants with median score of 15 (IQR 12–17). ICU and hospital LOS varied with median durations at 20 days (IQR 10–42 days) and 42 days (IQR 23–71 days), respectively. Demographic data for the total cohort are summarised in Table 1.

**Table 1:** Demographic and medical data for total (*n* = 235), dysphagic (*n* = 220), and non-dysphagic (*n* = 15) cohorts

Population variable	Total cohort (*n* = 235) (Median [range])	Dysphagic cohort (*n* = 220) (Median [range])	Non-dysphagic cohort (*n* = 15) (Median [range])
Age (years)	58 (21–97)	58 (21–97)	64 (35–89)
Number of intubations (*n* = 196)	1 (0–3)	1 (0–3)	1 (0–3)
Total duration of intubations (days) (*n* = 196)	14 (0–60)	15 (0–60)	8 (0–38)
Duration of tracheostomy (days) (*n* = 78)	31 (6–136)	31 (6–136)	60 (18–117)
Total duration of mechanical ventilation (days) (*n* = 196)	16 (0–164)	17 (0–164)	12 (0–116)
APACHE II score (*n* = 91)	15 (4–29)	15 (4–29)	12 (8–16)
ICU length of stay (days)	20 (1–164)	20 (1–164)	10 (2–156)
Hospital length of stay (days)	42 (1–293)	45 (5–293)	21 (1–194)

The majority of patients had several pre-existing co-morbidities at the point of hospital admission. These were most frequently hypertension (*n* = 110, 47%) followed by diabetes (*n* = 106, 45%). A comprehensive list of pre-existing co-morbidities can be found in Table 2.

**Table 2:** Past medical history data (*n* = 235)

Condition	*n* (%)
Hypertension	110 (47)
Diabetes	106 (45)
Cardiology	70 (30)
Gastroenterology	59 (25)
Other respiratory disease (incl asthma)	55 (23)
Mental health	54 (23)
Other	54 (23)
Other surgical	46 (20)
Renal	41 (17)
Hypercholesterolaemia	41 (17)
Obesity	40 (17)
Non-head & neck cancer	26 (11)
Other neurological condition	25 (11)
Sleep apnoea	23 (10)
Gout	19 (8)
Rheumatology	17 (7)
Endocrinology	17 (7)
Orthopaedic	17 (7)
Drug & alcohol	16 (7)
Stroke	13 (6)
Infectious diseases	12 (5)
Ophthalmology	11 (5)
Dyslipidaemia	11 (5)
Vascular	11 (5)
COPD	10 4)
Genetic disorder	8 (3)
Haematology	6 (3)
Dementia/cognitive impairment	4 (2)
Head & neck cancer	2 (1)
Progressive neurological disease	0 (0)

Hospital acquired co-morbidities were frequently observed across the cohort with the two most common pathologies being ICU-Acquired Weakness (*n* = 127, 54%) and delirium (*n* = 115, 49%). Additional complications were documented and are summarised in Table 3.

**Table 3:** Medical outcomes data (*n* = 235)

Medical outcome	*n* (%)
ICU-acquired weakness	127 (54)
Delirium	114 (49)
Cardiac event	39 (17)
Pressure injury	33 (14)
Failed extubation	27 (12)
Mortality	26 (11)
Neurological event	16 (7)
Laryngeal oedema	10 (4)
Pharyngeal oedema	3 (1)
None	11 (5)
Other: • Pulmonary embolus • Ventilator acquired pneumonia • Multi-organ failure • Sepsis • Acute respiratory distress Syndrome • Acute kidney injury • Pneumomediastinum • Severe lung fibrosis • Coagulopathy • Aspergillus pneumonia	137 (58)

Most participants were discharged directly home from acute care (*n* = 129, 55%), almost a quarter required inpatient rehabilitation (*n* = 56, 24%) and less were transferred to another acute facility (*n* = 21, 9%) or succumbed to mortality during the indexed admission (*n* = 26, 11%).

### Swallowing Outcomes

Prevalence of dysphagia on initial SLP assessment was 94% (*n* = 220) across the total cohort with the largest proportion (*n* = 106, 45%) exhibiting profound dysphagia (FOIS = 1) followed by those (*n* = 62, 26%) who were able to commence total oral nutrition requiring special preparation (FOIS = 5). Of the remaining 22% (*n* = 52) that were diagnosed as dysphagic on initial assessment, 7% (*n* = 16) were tube dependent with minimal attempts at oral intake (FOIS = 2), 8% (*n* = 19) were tube dependent with consistent modified oral intake (FOIS = 3), 3% (*n* = 6) were on a complete oral diet of a single consistency (FOIS = 4), and 5% (*n* = 11) were on a complete oral diet with specific food limitations (FOIS = 6).

Duration to initiate oral feeding was observed at a median of 19 days (IQR 11–44 days) from the time of ICU admission. Those who received dysphagia rehabilitation (*n* = 57, 24%), treatment was commenced at a median of 39 days (IQR 18–60 days). Dysphagia rehabilitation included a range of therapeutic strategies with active salivary swallows (*n* = 25, 44%) and effortful swallow (*n* = 22, 39%) most frequently prescribed. Table 4 summarises dysphagia rehabilitation utilised across the cohort.

**Table 4:** Dysphagia rehabilitation used across the cohort (*n* = 57)

Therapeutic strategy	*n* (%)
Ice chips	12 (21)
Salivary swallows	25 (44)
Effortful swallow	22 (39)
Masako manoeuvre	10 (18)
Mendelsohn	0 (0)
Chin tuck against resistance/shaker	4 (7)
Expiratory muscle strength training	6 (11)
Other: • Therapeutic oral trials (SLP led) • Above cuff airflow • McNeill dysphagia treatment programme	18 (32)

Resolution of dysphagia for the total cohort was achieved by the time of hospital discharge in 71% (*n* = 168) of participants with a median duration to recovery of 30 days (IQR 17–56 days). Enteral feeding was required in 87% of cases (*n* = 205) with a median duration of 22 days (IQR 12–48 days).

VFSS was conducted in 9% (*n* = 20) and FEES in 14% (*n* = 34) of the total cohort. High rates of airway invasion on fluids (PAS = 3–8) was observed on both videofluoroscopic (60%, *n* = 12) and endoscopic examination (*n* = 23, 68%). Furthermore, pharyngeal clearance was also an apparent issue with some degree of pharyngeal residue on either food or fluids evident in 65% (*n* = 13) on VFSS (BRS = 2–6) and 100% (*n* = 34) on FEES (Yale = 2–5). FEES also enabled assessment of secretion management with 26% (*n* = 9) demonstrating laryngeal penetration or aspiration of secretions (NZSS = 5–7) and 35% (*n* = 12) pharyngeal retention of secretions (NZSS = 1–4).

### Associations Between Demographic, Medical, and Swallowing Data

Several associations were identified between demographic, medical, and swallowing outcomes. Positive linear associations were observed between the duration to SLP assessment, commencing oral feeding, commencing dysphagia rehabilitation, dysphagia recovery and enteral feeding, and the duration of intubation, tracheostomy, mechanical ventilation, ICU, and Hospital LOS. Older participants were associated with a clinical presentation of more severe dysphagia on initial assessment; however, interestingly, younger patients exhibited greater duration to SLP assessment and commencement of oral intake. Furthermore, the presence and severity of dysphagia on initial SLP assessment was inversely correlated with critical care interventions and LOS. There was no association between the presence of dysphagia and APACHE II score (*Z* = − 1.855, *p* = 0.064) or prone ventilatory positioning (*r* = 0.436, *p* = 0.509). Demographic and medical outcome data for the dysphagic and non-dysphagic cohorts are presented in Table 1, and all swallowing outcome association data are summarised in Table 5.

**Table 5:** Associations between demographic, critical care, and swallowing outcomes (*n* = 235)

	Median (IQR)	Age	APACHE-II	Number of intubations	Intubation duration	Tracheostomy duration	Mechanical ventilation duration	ICU LOS	Hospital LOS
Presence of dysphagia: Z (*p*-value)	–	− 1.29 (0.197)	− 1.855 (0.064)	− 0.268 (0.789)	− 2.454* (0.014)	− 1.541 (0.123)	− 1.990* (0.047)	− 2.839** (0.005)	− 3.243** (0.001)
Duration to SLP assessment: r (*p*-value)	17 (10–36)	− 0.218** (0.000)	− 0.133 (0.209)	0.262** (0.000)	0.651** (0.000)	0.341** (0.002)	0.786** (0.000)	0.812** (0.000)	0.684** (0.000)
Dysphagia Severity: r (*p*-value)	2 (1–5)	0.143* (0.028)	− 0.034 (0.752)	− 0.136* (0.037)	− 0.262** (0.000)	− 0.203 (0.075)	− 0.362** (0.000)	− 0.42** (0.000)	− 0.396** (0.000)
Duration to initiate oral feeding: r (*p*-value)	19 (11–44)	− 0.130* (0.047)	− 0.003 (0.977)	0.295** (0.000)	0.480** (0.000)	0.518** (0.000)	0.715** (0.000)	0.788** (0.000)	0.725** (0.000)
Duration to commencing dysphagia rehabilitation: r (*p*-value)	39 (18–60)	0.098 (0.465)	− 0.073 (0.730)	0.035 (0.794)	0.392** (0.003)	0.487** (0.004)	0.739** (0.000)	0.672** (0.000)	0.598** (0.000)
Duration to recovery of dysphagia: r (*p*-value)	30 (17–56)	− 0.092 (0.237)	0.44 (0.726)	0.200** (0.01)	0.448** (0.000)	0.602** (0.000)	0.782** (0.000)	0.814** (0.000)	0.867** (0.000)
Duration of enteral feeding: r (*p*-value)	22 (12–48)	− 0.106 (0.151)	− 0.065 (0.574)	0.062 (0.397)	0.413** (0.000)	0.538** (0.000)	0.740** (0.000)	0.742** (0.000)	0.672** (0.000)

*Sign at 0.05; **Sign at 0.01

### Comparison to Previously Published Cohort

**Fig. 1:**
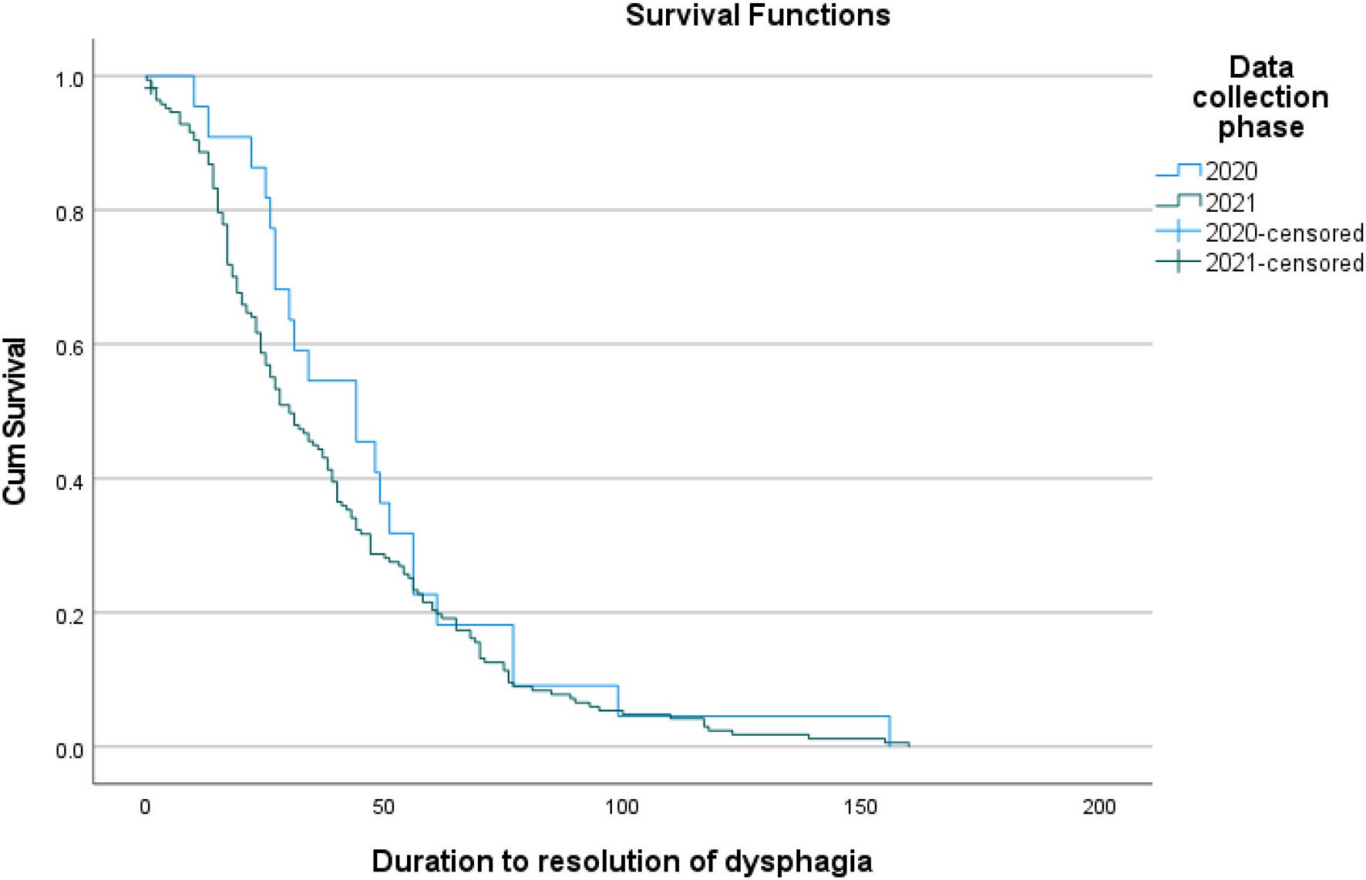
Kaplan–Meier survival curve comparing duration of dysphagia recovery

For the COVID-19 participant requiring ICU treatment and referred to SLP, the prevalence rate of dysphagia within the current study (94%, *n/N* = 220/235) was comparable (*p* = 1.000) to the prevalence rate cited in the authors’ earlier work (93%, *n/N* = 25/27). Dysphagia severity was analogous between the two cohorts (*Z* = − 0.889, *p* = 0.374) as was the trajectory of duration to dysphagia recovery as shown in the Kaplan–Meier survival curve illustrated in Fig. 1. Application of instrumental assessment was also similar across both time periods (19% vs 20%, *p* = 0.901).

Most demographic and critical care outcomes were comparable across the 2 time periods (*p* > 0.05); however, in the current study, participants were significantly younger (*Z* = − 2.169, *p* = 0.03), were more likely to have required tracheostomy (*r* = 7.153, *p* = 0.007), and had a shorter duration of ICU LOS (*Z* = − 2.389, *p* = 0.017). Further to this, overall participants were seen for SLP assessment of swallowing (*Z* = − 3.099, *p* = 0.002), and commenced oral feeding (*Z* = − 2.115, *p* = 0.034) earlier. The proportion of participants that demonstrated persistent dysphagia at the time of hospital discharge was greater in the current study versus the prior study (29% vs 19%); however, this was not statistically significant (*p* = 0.271).

## Discussion

At the time of submission, this is the largest multi-site and geographical prospective study to examine swallowing outcomes in the critically ill COVID-19 patient requiring ICU admission. Our study confirmed that the prevalence (94%) and severity of dysphagia (45% profound dysphagia) in the ICU COVID-19 patient who is referred to SLP remains disturbingly high. Further to this, the duration to commence oral intake and for dysphagia to resolve was associated with most critical care outcomes including duration of intubation, tracheostomy, mechanical ventilation, ICU and Hospital LOS, which is in line with other non-COVID critical care dysphagia literature [23–27]. Contrary to other non-COVID critical care populations, however, presence and severity of dysphagia were inversely associated with medical outcomes. Moreover, in more recent waves of the pandemic, hospitalised patients infected with SARS-CoV-2 appear to be younger and require tracheostomy more frequently, although their ICU LOS is shorter. Dysphagia was also more likely to persist beyond hospital discharge, aligning with known moderate to severe levels of persistent new physical disability following critical illness [28].

The concept that the evolution of the pandemic has seen the presence and severity of dysphagia being associated with *shorter* durations of intubation, mechanical ventilation, ICU, and Hospital LOS is an interesting finding. This potentially supports the hypothesis that the mere diagnosis of COVID-19 alone may be enough to increase to risk for dysphagia given the reduced duration of known risk factors. Given that COVID-19 is a disease initially affecting the aerodigestive tract [29], and the known intricate relationship between respiration and swallowing to optimise airway protection [30], this is not unreasonable. Moreover, there is a substantial body of evidence not only in COVID-19 but also other respiratory conditions that highlight dysphagia as a manifesting problem warranting diligent assessment and management [1–3, 5, 7, 31–36].

In the current study, patients who were younger also took longer to be seen by SLP and initiate oral intake. One potential theory is that with the known high rates of morbidity and mortality associated with COVID-19 and supported by the current study’s data showing that older participants had poorer APACHE-II scores and higher rates of mortality that younger patients took longer to commence oral intake because they were fortunate enough to survive. This contrasts with earlier work by the current authors which identified no association between age and swallowing outcomes [2] and the work by Regan et al. [6, 36] that describe increased age as a predictor for post-extubation oral intake status. This variability in published evidence suggests that age may not be the most reliable factor to consider when evaluating risk for dysphagia post-COVID-19.

The role of instrumental assessment between the two cohorts examined was surprisingly comparable. It is plausible to consider that this may have been due to higher numbers of COVID-positive patients being referred for SLP consultation and lack of clinician capacity to conduct instrumental assessments, or alternatively, an ongoing reflection of the hesitation to conduct additional instrumentation on infectious patients [37]. Whilst the application of Flexible Nasal Endoscopy (FNE) and Videofluoroscopic Swallowing Study (VFSS) in COVID-19 was initially stemmed due to concerns of airborne viral transmission, refinement of infection control practices has anecdotally seen a return of such procedures which substantiates the presence of dysphagia and enables more detailed assessment of laryngeal pathology as well as swallow function. Despite this, only few studies report on swallowing outcomes as defined by instrumental assessment in COVID-19 patients [10, 38, 39]. Osbeck Sandblom et al. [38] describe that on endoscopic examination, high proportions of impaired vocal fold movement in 76% and impaired swallowing in 96% of critically ill patients with COVID were observed. Webler et al. [10] applied VFSS in their COVID-19 cohort, enabling quantification of silent aspiration and use of instrumental assessment enabled diet/fluid upgrades and downgrades, ultimately increasing safety considerations in ongoing management. Further to these two studies, Boggiano and colleagues [40] confirmed high rates of laryngeal pathology in COVID-19 patients within the ICU describing that 63% had ≥ 1 clinically significant laryngeal pathology on FEES, which was higher compared to non-COVID comparison group. More information is required detailing the pathophysiology of swallowing impairment to inform timely swallow rehabilitation and optimise patient outcomes, including optimising safety and rehabilitation planning in this challenging population.

The higher rate of persistent dysphagia at the time of hospital discharge in the current cohort compared to the authors’ earlier study (29% vs 19%) is also noteworthy and is similar to those documented by Archer et al. [3] (also 29% persistent dysphagia) and Regan et al. [36] (27% persistent dysphagia). This may be reflective of the pressures to reduce hospital LOS during surges of hospital admission in line with waves or new variants of the pandemic. There are multiple factors that could have contributed to this; however, investigation of these were not the primary aims and were beyond the scope of this study.

### Strengths and Limitations

To the authors’ knowledge and at the time of submission, this study provides the largest international prospective multi-site project also covering the largest geographical area, reporting dysphagia prevalence and outcomes in the ICU COVID-19 population to date. Furthermore, study rigour was strengthened by the provision of clinician training in data collection and application of a data dictionary to ensure accuracy of outcome measurement. Despite this, limitations do exist. Not every patient admitted to the ICU was screened for presence of swallowing impairment; the study design implemented was intentionally pragmatic, with only those who were referred to SLP considered for study inclusion due to clinical capacity constraints. Whilst it is therefore possible that the prevalence rate of dysphagia identified in this study may be under-represented, it is reassuring that those patients who were referred to SLP for assessment were in fact appropriately referred. Furthermore, not every patient underwent instrumental swallowing assessment. Potentially related to this was also the low rate and variable range of rehabilitation techniques applied to those diagnosed with dysphagia. Consequently, treatment strategies and their subsequent association on recovery of swallow function described in this study should be interpreted with caution. Reasons for the low rate of instrumental assessment and prescription of rehabilitation again include the pragmatic observational nature of this study design as well as constraints to instrumental assessment access across facilities in line with individual pandemic site guidelines. Application of VFSS or FEES is encouraged in the future studies and would provide valuable data regarding the pathophysiology of swallowing impairment. Moreover, whilst the application of outcome scales to quantify and evaluate swallowing function does allow for consistency in rating, they do not directly inform on physiological deficits. Each of these scales applied in the current study were selected as they are recognised as simple tools that can be efficiently applied to objectify VFSS and FEES interpretation specific to penetration / aspiration and pharyngeal clearance. Future studies ideally should report on physiological swallowing parameters for both VFSS and FEES examinations.

## Conclusion

Dysphagia continues to be highly prevalent and persistent in the ICU COVID-19 patient and is strongly associated with critical care outcomes. However, as the COVID-19 pandemic with its variants continues to evolve, it appears that hospitalised dysphagic patients are younger. Additionally, whilst tracheostomy appears to be increasingly utilised, subsequent ICU but not hospital LOS is shorter. For those who exhibit dysphagia, duration to commence oral intake is more rapid, but less achieve complete dysphagia recovery by hospital discharge. These findings support the need for SLP in this critical care population as well as the need for continued multidisciplinary awareness to initiate SLP referrals in a timely manner. Furthermore, greater evidence on dysphagia pathophysiology is still required to guide efficacious swallowing rehabilitation in this complex population.

## Data Availability

All data from individual sites have been retained within a purpose-built password-protected REDCap database, saved on a secure password-protected shared drive housed on the Sydney Local Health District Speech Pathology server. All data may be downloaded via a secure encrypted link to which only the listed authors have access to.
